# Can sugar taxes be used for financing surgical systems in Nigeria? A mixed-methods political economy analysis

**DOI:** 10.1093/heapol/czae021

**Published:** 2024-03-29

**Authors:** Martilord Ifeanyichi, Cyril Dim, Maeve Bognini, Meskerem Kebede, Darshita Singh, Obinna Onwujekwe, Rachel Hargest, Rocco Friebel

**Affiliations:** Global Surgery Policy Unit, LSE Health, London School of Economics and Political Science, Cowdray House 1.12, Houghton Street, London WC2A 2AE, United Kingdom; Health Policy Research Group, Department of Pharmacology and Therapeutics, University of Nigeria Enugu Campus (UNEC), Enugu, Nigeria; Department of Obstetrics and Gynaecology, Faculty of Medical Sciences, College of Medicine, University of Nigeria, Ituku-Ozalla, Enugu, Nigeria; Global Surgery Policy Unit, LSE Health, London School of Economics and Political Science, Cowdray House 1.12, Houghton Street, London WC2A 2AE, United Kingdom; Global Surgery Policy Unit, LSE Health, London School of Economics and Political Science, Cowdray House 1.12, Houghton Street, London WC2A 2AE, United Kingdom; Global Surgery Policy Unit, LSE Health, London School of Economics and Political Science, Cowdray House 1.12, Houghton Street, London WC2A 2AE, United Kingdom; Health Policy Research Group, Department of Pharmacology and Therapeutics, University of Nigeria Enugu Campus (UNEC), Enugu, Nigeria; Global Surgery Policy Unit, LSE Health, London School of Economics and Political Science, Cowdray House 1.12, Houghton Street, London WC2A 2AE, United Kingdom; School of Medicine, University Hospital of Wales, Cardiff CF14 4XN, United Kingdom; Royal College of Surgeons of England, London, United Kingdom; Global Surgery Policy Unit, LSE Health, London School of Economics and Political Science, Cowdray House 1.12, Houghton Street, London WC2A 2AE, United Kingdom; Center for Global Development Europe, London SW1P 3SE, United Kingdom; Department of Health Policy, London School of Economics and Political Science, London WC2A 2AE, United Kindom

**Keywords:** Global surgery, health policy, health financing, innovative financing, Nigeria

## Abstract

This study determined the feasibility of investing revenues raised through Nigeria’s sugar-sweetened beverage (SSB) tax of 10 Naira/l to support the implementation of the National, Surgical, Obstetrics, Anaesthesia and Nursing Plan, which aims to strengthen access to surgical care in the country. We conducted a mixed-methods political economy analysis. This included a modelling exercise to predict the revenues from Nigeria’s SSB tax based on its current tax rate over a period of 5 years, and for several scenarios such as a 20% ad valorem tax recommended by the World Health Organization. We performed a gap analysis to explore the differences between fiscal space provided by the tax and the implementation cost of the surgical plan. We conducted qualitative interviews with key stakeholders and performed thematic analyses to identify opportunities and barriers for financing surgery through tax revenues. At its current rate, the SSB tax policy has the potential to generate 35 914 111 USD in year 1, and 189 992 739 USD over 5 years. Compared with the 5-year adjusted surgical plan cost of 20 billion USD, the tax accounts for ∼1% of the investment required. There is a substantial scope for further increases in the tax rate in Nigeria, yielding potential revenues of up to 107 663 315 USD, annually. Despite an existing momentum to improve surgical care, there is no impetus to earmark sugar tax revenues for surgery. Primary healthcare and the prevention and treatment of non-communicable diseases present as the most favoured investment areas. Consensus within the medical community on importance of primary healthcare, along the recent government transition in Nigeria, offers a policy window for promoting a higher SSB tax rate and an adoption of other sin taxes to generate earmarked funds for the healthcare system. Evidence-based advocacy is necessary to promote the benefits from investing into surgery.

Key messages

- The Nigerian sugar tax policy has a potential to generate ∼36 million USD annually and 190 million USD during the period of 5 years, which is ∼1% of the total cost of the national surgical plan.
- Implementation of the World Health Organization (WHO) recommended 20% ad valorem tax could generate ∼85 million USD in the first year, ∼49 million USD more than under the currently implemented tax rate.
- Stakeholders should support the Government to review the policy towards the WHO recommended 20% ad valorem rate and ring-fencing the revenues for health.
- Despite the existence of a national surgical plan in Nigeria, intense evidence-driven advocacy and engagement within and without the health space is still needed to elevate the place of surgery in national resource allocation priorities.

## INTRODUCTION

Five billion people lack access to safe, timely and affordable surgery in the world. Of the 313 million procedures performed annually, only 6% occur in the poorest countries, where the greatest need lies (Meara *et al*., 2015). In Nigeria, 166 surgeries per 100 000 population are performed each year compared to the recommended volume of 5000 surgeries per 100 000 population, leaving most people suffering from surgical diseases unable to receive the care they need (Federal Ministry of Health and Social Welfare of Nigeria, 2019). For those that access surgical interventions, >65% of Nigerians experience impoverishment and catastrophic health expenditure (Federal Ministry of Health and Social Welfare of Nigeria, 2019). Spurred by the 2015 World Health Assembly resolution 68:15, mandating countries to include emergency and essential surgical care as a core component of Universal Health Coverage (UHC) (Federal Ministry of Health and Social Welfare of Nigeria, 2019), the Nigerian government launched its first National Surgical, Obstetrics, Anaesthesia and Nursing Plan (NSOANP) in 2019. It provides a situational analysis of surgical care in Nigeria, a roadmap for improvement, and an in-built monitoring and evaluation mechanism. With a full implementation cost of ∼$17 billion, the plan seeks to scale-up surgical care to 75% population coverage, and 50% coverage for children <15 years of age between 2019 and 2023. Specific targets include increasing surgical, anaesthesia and obstetric specialists to 5 per 100 000 population and paediatric surgeons to 0.25 per 100 000 children under the age of 15 years, increasing surgical volume by 100%, training family physicians and general duty doctors to provide Bellwether procedures and essential children’s surgery package, and training general duty doctors and specialists to provide anaesthesia at secondary and primary level hospitals. The Nigerian surgical plan is the first across low- and middle-income countries (LMICs) to explicitly include paediatric and adolescent surgical care.

The concept of national surgical plans in its current format was popularized by the 2015 Lancet Commission on Global Surgery (Meara *et al*., 2015). The commission recommended a context-specific plan, embedded within the broader national health policy, and structurally mirroring the broader health systems building blocks (Kebede *et al*., 2023). Several countries have, like Nigeria, developed and launched national surgical plans, including Zambia, Tanzania, Madagascar, Senegal, Namibia and Zimbabwe, and several others have committed to the process. Despite the optimism that have greeted these developments (Citron *et al*., 2019), the implementation of national surgical plans has been slow, at least partly due to poor financial resource allocation. Given that most countries in sub-Saharan Africa still allocate to health in their budgets <15% stipulated by the Abuja Declaration (Olalere and Gatome-Munyua, 2020) (e.g. Nigeria spent 4%, or $960 million, in 2019) (Seyi-Olajide *et al*., 2021b), experts have called for innovative financing mechanisms, including sin taxes on alcohol, cigarettes and sugar-sweetened beverages (i.e. soft drinks, or SSBs) to fund the NSOANP (Ifeanyichi *et al*., 2021; Seyi-Olajide *et al*., 2021a).

Sin taxes are excise taxes imposed on goods or services deemed harmful to public health (World Health Organization, 2022). They aim to reduce the consumption of these products, raise extra revenues for governments and improve overall population health (Miracolo *et al*., 2021). These may be specific (fixed amount per unit of the product) or ad valorem (a fixed percentage of the value of the product). Evidence from high-income countries indicates that health taxes can be highly effective (Wright *et al*., 2017), yet these are significantly underutilized policy interventions for advancing public health (Marten *et al*., 2022). In Latin America, evaluations have shown that a levy of 1 Peso/l of SSBs introduced in Mexico in 2013 reduced SSB consumption by ∼12% and provided the Mexican Government with an additional 2.6 billion USD within the first 2 years (James *et al*., 2020; World Health Organization, 2023). In sub-Saharan Africa, studies on the impact of sin taxes have focused mostly on South Africa, which, in 2018, had introduced a variable tax of 0.021 ZAR (∼$0.15) for every gram of added sugar above the initial threshold of 4 g/100 ml (Stacey *et al*., 2019). The SSBs tax has been associated with a 32% reduction in volume of soft drinks consumed among lower socioeconomic urban households and additional revenues of 5.8 billion ZAR over the first 2 fiscal years of the policy (Hofman, 2021). While Zambia also introduced a 3% health tax on SSBs in 2018 (Mukanu *et al*., 2021), the adoption across other LMICs of sub-Saharan Africa remains low, despite favourable evidence from political and economic landscape analyses (Abdool Karim *et al*., 2021; Thow *et al*., 2021).

On 31 December 2021, Nigeria became the second LMIC in sub-Saharan Africa to impose levies on SSBs, with the enactment of a new finance law stipulating a specific excise tax of 10 Naira (N) per litre of non-alcoholic, carbonated sweetened beverages (Adedeji and Iii, 2022). The policy implementation became effective on 1 June 2022. This study aimed to investigate policy opportunities for financing the surgical system strengthening initiatives of Nigeria using a political economy analysis and inform strategies to optimize positioning of surgical care in the sugar tax policy. Specifically, underpinned by the overarching question, ‘How and to what extent can sin taxes on SSBs support NSOANP implementation in Nigeria?’, this study modelled the potential revenues derivable from implementation of the sin tax; explored the barriers and facilitators of a successful policy implementation; assessed the opportunities of channelling the raised finances to health care specifically; and investigated the possibilities of dedicating the resources to surgery specifically.

## METHODS

### Study design and theoretical framework

We conducted a mixed-methods political economy analysis (PEA), a methodology used widely in the study of (international) development programmes, to understand determinants as well as the beneath-the-surface forces that inform the design of programmes, and to understand why programmes failed (DFID, 2009). It involves exploration of ‘how things work’ in the local environment to identify entry points for ‘politically smart interventions’ (DFID, 2009). While PEAs are most often qualitative in nature, World Bank experts insist that best quality PEAs deploy a range of quantitative and qualitative tools to explore the underlying political drivers, constraints and opportunities for change (Kossoff, 2015). We designed a quantitative model to assess the financing potential for NSOANP inherent to the SSB in Nigeria, combined with qualitative components that include document analysis and in-depth interviews with key stakeholders to investigate the feasibility of leveraging sugar tax revenues to provide earmarked funding for enhancing surgical care.

PEA had been conducted around soft drink taxes across several countries in Africa, including Botswana, Namibia, Kenya, Zambia, Rwanda, Tanzania and Uganda (Thow *et al*., 2021). Similar to the previous work, our study derives theoretical underpinnings from an integration of Campbell’s theories on the role of ideas and institutions (Campbell, 1998) and Luke’s third dimension of power in Luke’s three dimensions of power in PEA (Dowding, 2006). This approach therefore involves the exploration of ‘ideas and paradigms, institutional context, stakeholder interests and power’. ‘Ideas’ are perceptions, concepts and theories—obvious or subtle—that underpin policy debates while ‘institutions’ refer to structures and agencies as well as formal and informal rules that guide engagements and decision processes (Thow *et al*., 2021). ‘Stakeholder interests’ refer to the objectives pursued by different players and the factors they deem as important, while ‘power’ captures which actors influence decisions and mechanisms of achievement (Thow *et al*., 2021).

### Estimating potential revenues from SSB-taxes

In line with the existing evidence on the mechanism of sin taxes, our study assumed that an increase in tax rate will lead to an increase in price and therefore a drop in demand of SSBs. Therefore, the additional revenues from the newly imposed sin tax depend on the magnitude of the change in demand. The revenues raised from the new tax was estimated using a mathematical model developed in Microsoft Excel (*y* = 10x, where *y* = revenues in Naira; *x* = quantity of SSBs consumed in litres after accounting for the drop in demand in response to the new tax).

Data used to populate the model are shown in Supplementary Material 1. The extent to which the increase in tax will affect the retail price depends on the ‘pass-on rate’*—*the proportion of the tax that is passed on to the consumers in the form of higher retail prices. The base model assumed a 100% pass on rate, which has been used in previous studies in similar contexts, including Zambia and South Africa (Hangoma *et al*., 2020).

The extent to which the increase in price affects the consumption of SSBs depends on the ‘own-price elasticity’, which is the measure of the responsiveness of the demand for a commodity to changes in the prices of the commodity. We used an ‘own-price elasticity’ of −1.30, based on findings on SSB elasticities derived from a comprehensive meta-analysis (Hangoma *et al*., 2020).

In the absence of the policy, the model assumed a baseline consumption of SSBs in Nigeria of 1 638 536 886 litres in 2022. This estimate was provided based on 2021 consumption data by Euromonitor (Euromonitor, 2021; Lawal, 2022). The 2021 data were adjusted for population growth of Nigeria, using data from United Nations World Population Prospects (WPP) 2022 (World Population Prospects—Population Division—United Nations, 2022). In the absence of reliable data on the average prices (per litre) of the SSBs in Nigeria, we did not attempt to estimate the change in prices of soft drinks with the introduction of the policy, but we assumed the percentage equivalent of the 10 Naira/l to be 7%, as had been widely reported in the media. Change in demand of soft drinks was then estimated by dividing the change in price by its own price elasticity.

After obtaining estimates for the base year, the estimates were extrapolated for 4 years (2023–2027), by adjusting the consumption rate by population growth projection data from the United Nations WPP (World Population Prospects—Population Division—United Nations, 2022). Revenue projections were derived by applying the estimated consumption levels in litres to the tax rate. The potential average annual revenue was then estimated from the total derivable during the 5-year period.

The annual revenue estimates were compared to the annual costs of the NSOANP implementation to determine the proportions of the implementation plan that could be derived from the taxes. As the NSOANP was already completing its 4th year and there was no evidence of tangible progress in the implementation, our analysis assumed a fresh 5-year mandate starting from 2022. As such the costs were recalibrated from 2018–2023 to 2022–2027. As the original planning had assumed an annual inflation rate of 12%, the 2022 cost was adjusted using actual inflation data, while 2023–2027 costs were adjusted using the latest inflation projections (Central Bank of Nigeria, 2022; O’Neill, 2023)

### Sensitivity analyses

We conducted sensitivity analyses to assess the uncertainties around the model input variables and how they affect the results. A deterministic sensitivity analysis (DSA) was performed, which involved changing the input parameter values [i.e. changing the value of one parameter while keeping all others constant (univariate DSA)]. Each parameter was adjusted by 20% (±). The results of the DSA were presented in a tornado diagram. Scenario analyses were conducted using different tax rates (per litre) of 20 Naira (14%), 50 Naira (35%), 100 Naira (70%) and an ad valorem rate of 20% recommended by the World Health Organization (WHO). We also modelled scenarios with different baseline consumption figures, as derived from document analysis, including (i) 12.4 billion litres, suggested by Statista, a global data organization (Itsibor, 2022; Statista, 2022), and (ii) 400 million litres, widely quoted by civil society organizations (CSOs) (Bankole, 2022). In addition, we modelled a scenario of 33% change in retail prices of soft drinks with the 10 Naira/litre tax rate as reported by local newspapers (Bailey, 2022). All revenue figures were converted from Naira to USD using the mid-2022 (June 30) Central Bank of Nigeria exchange rate (Central Bank of Nigeria, 2022).

### Qualitative data collection

To understand the immediate and wider policy and legal landscape, we reviewed relevant legislative acts, policy frameworks, and executive orders issued in Nigeria. We reviewed print and electronic media reports, interviews, comments and commentaries to appreciate the discourses. Using a predesigned data extraction form, we extracted information related to the key themes of ‘laws, powers, ideas, institutions and stakeholder interests’, as they relate to opportunities and/or barriers for financing of surgery from the SSB tax revenues.

Further insights were obtained from in-depth interviews of key stakeholders and subject-matter experts. Interview respondents were purposively selected, based on the researchers’ knowledge of the context, document reviews and snowballing. The selection aimed to capture policymakers, surgery practitioners, global surgery scholars and champions, health policy and financing experts and CSOs. In addition, medical practitioners from non-surgery specialities were included specifically to explore the chances of non-surgery practitioners prioritizing surgery over their own specialities. Identified (potential) participants were contacted via email, with the study information form, study protocol, ethical clearance and a formal request to participate. An invitee who did not respond within 2 weeks or who indicated unavailability due, for instance, to time constraints were replaced with another invitee who had a comparable professional profile. A total of 10 stakeholders were interviewed (details provided in Supplementary Material 2). Semi-structured interviews were conducted using a predesigned interview guide (presented in Supplementary Material 3). Question sets were organized in multi-staged clusters around: (i) successful implementation of the policy; (ii) possibilities of earmarking the raised funds to healthcare; (iii) possibilities of channelling funds wholly or partially to the financing of surgery, given competing interests within the health system. In addition, the interviews were used for triangulation and clarification of findings from document reviews. Each interview was conducted and recorded on zoom, lasting between 60  and 90 min.

All interviews were conducted in February 2023 by M.I., a research assistant professor of health financing. A qualified medical doctor with clinical practice experience in Nigeria, M.I. has a PhD in global surgery economics and possesses extensive experience in qualitative research methods. He had no clinical or academic relationships with the study participants prior to the study. While his in-depth understanding of the Nigerian healthcare and political systems facilitated a finer appreciation of the information from the respondents, as a researcher, he made conscious effort to insulate the conversations from personal biases.

### Qualitative data handling, analysis and reporting

Recordings were transcribed verbatim, and participants were anonymized and assigned code numbers prior to the analysis. The interviewer reviewed the transcripts for accuracy. Nvivo 12 (QSR International Pty Ltd, Victoria, Australia) data management software was used for handling and conducting thematic analyses (Braun and Clarke, 2006). To optimize analytical rigour, we employed multiple coders with diverse backgrounds, including two clinician-health economists with practice and qualitative research experience in low-income settings (M.K., D.S.) and one law expert with advanced training in international health policy, including qualitative research (M.B.) While maintaining the predefined PFA concepts as the overarching themes, each transcript was first coded independently by at least two coders, and through an iterative process of reading, re-reading and discussion, the team consensually identified and coded emerging subthemes, while maintaining a record of the decisions and evolution in the analytical process (audit trail). By identifying patterns and connections, related concepts were then linked together (axial coding). Initial results were presented to a broader group of researchers for a consensus on the thematic matrix. In addition, the study findings were presented to a cross-section of senior health managers from Nigeria for validation. Data from the document review and interview analysis were combined and reported based on the PFA thematic framework. Selected quotes were also included for illustration purposes. Quotes from respondents were anonymized while those from the media were not. The reporting followed the Consolidated Criteria for Reporting Qualitative Research (COREQ), which is a 32-item checklist for interviews and focus groups (Booth *et al*., 2014).

## RESULTS

### Revenue projections from SSB taxes in Nigeria

Using the base model, the SSB policy in Nigeria has the potential to generate 35 914 111 USD in year 1, and an aggregate revenue potential of 189 992 738 USD during the period of 5 years (see Table 1). Based on the total 5-year-adjusted cost of the NSOANP (20 billion USD), the current revenue potential of Nigeria’s SSB tax accounts for ∼1% of the total cost of the NSOANP implementation.

**Table 1.** Potential revenues derivable from the sin tax in the first 5 years

**Year**	**Amount (in USD)**
1	35 914 111
2	36 779 079
3	37 657 878
4	38 548 805
5	41 092 865
**Total**	**189 992 738**

**Figure 1.**
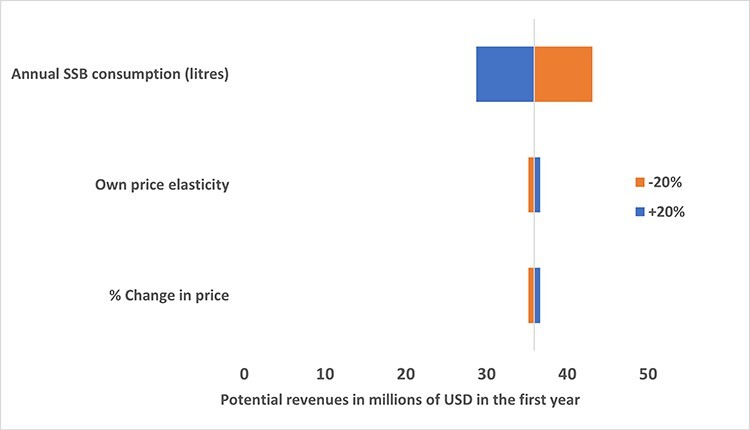
Results of the univariate deterministic sensitivity analysis

According to the univariate DSA, changes in value used for baseline consumption of sugar drinks had the greatest impact (see Figure 1). A 20% decrease in the value resulted in 28 731 289 USD and a 20% increase generated 43 096 934 USD in the first year. Similar adjustments to the retail price and own price elasticities (each) resulted in revenues of 36 633 184 USD and 35 195 039 USD, respectively.

The results of the re-estimation of our prediction model according to varying scenarios are presented in Table 2. The greatest revenue potential was obtained in scenario with baseline soft drink consumption of 12 billion litres, resulting in a potential of 273 130 037 USD. A five-factor increase in the current tax rate (i.e. tax rate of 35%) could generate 107 663 315 USD in the first year, while a ten-factor increase (i.e. tax rate of 70%) will drop the potential revenues to 35 558 526 USD as the drop in consumption is expected to outstrip the residual consumption. Implementation of the WHO recommended 20% ad valorem tax could generate 84 787 330 USD in the first year, 49 228 804 USD more than under the currently implemented tax rate.

**Table 2.** Potential SSB tax revenues in year 1 under different model assumptions

**Scenario**	**Amount (USD) in year 1**
Primary model	35 914 111
Baseline consumption of 12.4 billion litres	273 130 037
Baseline consumption 400 million litres	2 542 535
A price increase of 33% from a 10NGN/litre tax (7%)	22 559 909
A tax rate of 14%	64 637 499
A tax rate of 35%	107 663 315
A tax rate of 70%	35 558 526
A tax rate of 20% (recommended by WHO)	84 787 330

### The policy landscape of SSB tax

The findings from the document analysis and the in-depth interviews are summarized further and organized by theoretical themes. Stakeholder interests and power have been combined.

#### Ideas

Most respondents emphasized that health financing in Nigeria is inadequate and fails to respond to the population’s needs. The Federal Government’s budgetary expenditure is significantly low, having remained stagnant at <6% of total annual budget in many years. The limited breadth and depth of the national health insurance coverage also fails to offer adequate financial protection to the population. As a result, healthcare services are predominantly funded through out-of-pocket payments by patients at point of use.

Participants identified two possible strategies to raise revenue for health. They proposed to expand health insurance coverage to the informal sector workers, and to impose earmarked taxation on luxury goods and unhealthy substances. Therefore, introduction of SSBs taxes in Nigeria was particularly welcomed by most respondents. This innovative financing mechanism was seen as serving the double purpose of raising excise duties and revenues and, simultaneously, contributing to the prevention of non-communicable diseases (NCDs) by discouraging excessive consumption of sugary drinks.

However, one respondent questioned the propriety of sugar taxes in the Nigerian context, arguing that obesity is still predominantly a disease of the West, and that tax will affect the poor (e.g. menial workers) who survive on SSBs in their daily endeavours. Another insisted that sin taxes are not effective as people still smoke despite all the campaigns against smoking.

*For cigarettes, they will say it is harmful to health and they die young, has it discouraged anyone from smoking? There is a tax on it but people still smoke. If people quit, they quit not because of cost of product they are buying because ten naira will not change their lives. But they quit because it is a determined effort to quit taking…(MP4)*

Most of the interviewees ruled out the possibility of allocating the funds to the surgical system alone. Although they agreed on the value of the NSOANP in drawing the attention of policymakers to surgery, it still enjoys minimal recognition within the Nigerian health space. Interviewees argued that ‘every department in Nigeria needs money, they need so much money’ and surgical care is not perceived as a high-priority area. It is overshadowed by NCDs, given their close correlation with SSB consumption, as well as public health and infectious diseases, which have dominated the agenda of the Federal Government and international donors. Furthermore, in line with a horizontal ‘health systems strengthening’ approach, increasing attention has been drawn to primary healthcare, expanding the national health insurance coverage, and reinforcing healthcare infrastructure.

Interviewees shed light on key steps to be adopted by the surgical community to favour the channelling of resources to surgery. Advocacy plays a paramount role in persuading decision-makers. Advocates for surgery must delineate a compelling narrative, clearly communicating how surgery is closely tied to other priorities on the political agenda. For instance, supporting the allocation of sin tax revenue towards the national health insurance coverage: this would enable patients to access a wider selection of health services, including essential surgery, without incurring catastrophic expenditure. Additionally, basic surgical procedures should be seen as a core component of the medical services to be delivered at primary healthcare level. Importantly, all claims ought to be supported by research and data, proving the surgical burden experienced by the Nigerian population alongside the feasibility and cost-effectiveness of surgical interventions. Given the complexity and breadth of the surgical system, one interviewee suggested that ‘key entry points’ must be defined, to facilitate the allocation of funds in line with identified priorities.

#### Institutions

The federal structure of Nigeria’s 36 States and Federal Capital Territory (FCT) and the power dynamics between the national and sub-national governments was identified as having significant implications for managing the socio-political affairs of the country, including resource mobilization and resource allocation processes. Furthermore, the presidential system in Nigeria implies that the National Assembly (under considerable political influence of sub-national governments) makes laws while powers of policy formulation and execution of laws reside with the executive arm of the government (in this case, the Ministries of Finance and Health). These arrangements set-up multiple and complex potential facilitatory or inhibitory points, and therefore targets of advocacy by various stakeholders and interest groups, including CSOs, medical groups, international development partners and academic groups. Meanwhile, our study shows that advocacy engagements are not only between the government actors and external parties but also among government actors.

*So, the Ministry of Finance is really the one that was driving these because it appeared in the Finance Act. We have done our fair share of advocacy [working] with civil society groups, the Ministry of Health indicating why it’s important to do this…(PM1)*

The Nigerian constitution recognizes two categories of ‘items’ whereby items on the concurrent list (such as Health and Education) are joint responsibilities of the national and state governments while items on the exclusive list (such as Customs and Excise Duties, Defence, and Economy) are singular responsibilities of the national government. The sugar taxes are collected directly from the manufacturers and importers by the Customs Department and domiciled in a single national pool managed by the Federal Inland Revenue Service (FIRS), an agency of the Ministry of Finance. Revenues collected by FIRS are divided monthly among the national and sub-national governments. Moreover, the Finance Act did not explicitly ring-fence the revenues to be generated for health. Most of the respondents strongly challenged this approach fearing that the revenues could easily be lost in the national pool as they are diverted to other national needs, citing earlier experience with tobacco tax. Instead, respondents suggested pooling sugar tax revenues separately and amending the Act to secure the funds exclusively for healthcare, citing successful intervention funds like the defunct Petroleum Trust Fund which funded critical national infrastructure and the Tertiary Education Trust Fund financing infrastructure and manpower development at the higher institutions.

Our respondents identified two key relevant policy instruments in the broader context of health financing in Nigeria, including the National Health Act of 2014 that established the Basic Health Care Provision Fund (BHCPF), which is financed from at least 1% of the consolidated revenues of the Federation; and the National Health Insurance Authority (NHIA) Act (2022) which provides for mandatory health insurance in Nigeria and created the Vulnerable Group Fund. Some suggested channelling the benefits of the new Finance Act into these, especially after an attempt to raise revenues for the Vulnerable Group Fund through telephone call charges was shut down mid-2022 following public opposition. Within the context of surgery, a respondent identified the development NSOANP, creation of a budget line for NSOANP implementation in the annual budget of the Federal Ministry of Health and Social Welfare (FMOHSW) and the creation of a desk office for NSOANP at the FMOHSW as indicators that the government is desirous of prioritizing surgical systems. The respondent opined ‘It is doable’ to channel the sugar tax resources to NSOANP implementation with adequate stakeholder engagement.

Furthermore, we found three other less tangible but equally critical contextual elements. First, the Finance Act is reviewed yearly, and this provides opportunities to push for and correct the important shortcomings identified by the respondents. In addition, the importance of behind-the-door engagement came out strongly from the study as a powerful tool in advancing any agenda in Nigeria, beyond the public and media advocacy efforts. Lastly, most of the respondents emphasized ‘corruption’ as an institutional problem that must be checked for the sugar tax policy to succeed. They feared that the funds could ‘disappear into private pockets’ or ‘you will see part of that money in America, UK’, and made references to the case of ‘…pension where people’s money was all gathered together and disappeared’.

### Stakeholder interests and powers

Interviewees identified a multitude of state, non-state and private actors who influence the design and implementation of the sin tax policy. The FMOHSW actors conceived the idea as a means to diversify financing for health while addressing NCDs and sold the idea to the Ministry of Finance (PM1). The Ministry of Finance bought into and championed the development and implementation of the policy as a tool to alleviate wider fiscal space constraints in Nigeria. Local politicians saw the opportunity to raise more revenue for their individual States. The CSOs strongly campaigned in support of the policy primarily for reducing the risk of NCDs in Nigeria. Industry actors were opposed to the policy, insisting that the policy would harm broader government revenues and lead to loss of millions of jobs, but our respondents opined that the opposition from the industry stemmed purely from commercial interests given the financial disadvantages they might face as the result of reduced consumption of their products (CSO1).

*We know, for instance, that the food and beverage sub-sector contributes about 38% of the manufacturing contribution to Gross Domestic Product (GDP) and 22.5% of the jobs created, employing 1.5 million persons. We have a feeling that this is the kind of sector we need to guide against any negative thing falling there…It is what has been described by some who have commented on it as ‘pennywise and pound foolish’ in a sense. If you look at a revenue gain of N81 billion between 2022 and 2025, you compare this with a possible revenue loss of N142 billion in Value Added Tax (VAT) and N54 billion in Company Income Tax (CIT) over this period, I think it would be a better idea to actually allow the sector to continue on its growth pathways*.[Mr Ajayi-Kadir, Director-General, Manufacturers Association of Nigeria] (Olaoluwa, 2022)

Within the clinical and public health medical community, we found evidence of subtle inter-professional rivalry. Practitioners from different specialities were proponents of the policy to tax unhealthy habits while generating revenue for the health sector; however, they held significantly diverging views as to where the money should be invested with most strongly advocating for investments in their respective areas of practice.

Luke’s theory on the three dimensions of power (Dowding, 2006) provides an opportunity to visualize the power dynamics within the policy space, in the light of the findings from the respondents. The national government was the powerful actor identified as it possesses the ultimate ‘decision-making power’. This is on account of the role of the National Assembly as the law-making institution and functions of the executive arm through the Ministry of Finance which spearheads the policy. The representatives of the businesses exerted ‘non-decision-making power’ through backdoor negotiations, sponsorship of events which were used to prevent further escalation and implementation of the policy leading to restriction in government action.

*We are sure that there is a lot of lobbying, you know there is a lot of indoor discussion because they [The beverage Industry] can easily get a seat at the table .. (CSO2)*

The CSOs, more prominently the NASR (Nigerian Alliance for Sugar Reduction) and the medical community are also exerting considerable influence by formally organizing and setting up advocacy platforms. They utilize the media to frame the taxes as promoting healthy behaviours and an opportunity to redistribute wealth by largely taxing the rich. They are working to increase societal understanding of the taxes with increasing their ‘ideological power’, to produce evidence to support the implementation and further increment of the taxes and influence the decision by policy makers to ring-fence the money generated for healthcare services.

## DISCUSSION

Sin taxes have been proposed as possible levers to generate earmarked funding for healthcare systems, though their implementation is often faced by significant political, economic, legal, cultural, and social challenges. Multi-sectoral demands over the generated revenues make it difficult for policymakers to redistribute resources in a way that maximizes health gain. Given recent developments in Nigeria, including the introduction of an ambitious plan to transform population health through improved surgical services, we conducted a political economy analysis of the financing of the Nigerian NSOANP using funds from the recently introduced SSB tax. Findings from our modelling showed that the current tax rate of 10 Naira/litre can generate ∼35 million USD annually, possibly contributing 1% of the annual investment required for implementation of the NSOANP. At the current rate, the SSB tax appears insufficient to finance the NSOANP. Moreover, there remain major threats of diversion of the resources generated towards other national needs outside the healthcare sector. Even if the funds were successfully ring-fenced for health, the intra-health system forces influencing the allocation of resources are unlikely to favour surgery.

Sin taxes are often described as offering ‘win–win’ situations as they promote public health while simultaneously raising extra funds for investments in healthcare. Achieving these requires applying a rate that is high enough to disincentivize the consumption of the product and still raises resources from the continued consumption. Our findings suggest that the rate currently set in Nigeria remains too low to either discourage the consumption of soft drinks or raise significant resources for the government. While revenues generated through the SSB tax in Nigeria are significantly larger than those reported for Zambia (Hangoma *et al*., 2020), our base estimates are significantly lower than the Manufacturers Association of Nigeria (MAN) estimated Federal Government of Nigeria revenue of 193 million USD (vs 111 million USD) in the first 3 years (Olaoluwa, 2022). While there are no immediately available insights into the methodology employed by MAN, their estimates are also significantly lower than the revenues reported in Mexico within the first 2 years (James *et al*., 2020). The low tax level is itself a reflection of the politics that shaped the passage of the SSB tax law, especially the strong opposition from influential commercial stakeholders who often project sin taxes as impoverishing and anti-people, while in fact pursuing corporate interests (Olaoluwa, 2022). This aligns with experiences from other jurisdictions. For instance, the first attempt to introduce an SSB tax in Colombia in 2016 failed due to opposition from the industry (Garcia *et al*., 2017). In Zambia, the SSB tax law narrowly escaped the Colombian experience after the initial proposal of 25% had been reduced to 3% (Hangoma *et al*., 2020). It is noteworthy that several empirical and modelling studies on the impact of SSB taxes on employment, though mostly from high-income settings, have found no evidence in support of this common unemployment argument put forward by commercial stakeholders (Powell *et al*., 2014; Lawman *et al*., 2019; Marinello *et al*., 2021).

Setting the correct tax rate is crucial to the success of such programmes. Moreover, specific taxes such as the current Nigerian sin tax regime are prone to progressive impact dilution due to inflation, such that overall price inflation drowns the price sensitivity effect of the tax overtime. It therefore renders the policy ineffective, at least from the direct health impact perspective. However, ad valorem taxes in contrast are immune to such inflationary effects as the tax is always a percentage of the baseline prices. In line with WHO recommendations, we encourage an increase in the tax rate, and a shift to an ad valorem rate of at least 20% to ensure the sustainability of both the health and revenue benefits of the policy. According to our modelling, aligning the SSB tax in Nigeria with these recommendations would raise an additional revenue of up to 50 million USD per year compared to the current rate. This shift is currently being considered by the Ministry of Finance of Nigeria and based on our findings we would encourage all stakeholders to support the initiative as it will lead to substantial population health benefits and increase the fiscal space for investments into the healthcare system.

While the launch of the NSOANP has generated momentum around the need for surgery and the available surgical capacity in Nigeria, positioning surgery in a vantage position in the allocation of sin tax resources requires further innovative approaches. National Action on Sugar Reduction (NASR)—a coalition of several CSOs—for instance, has contributed significantly to the launch of the SSB tax in Nigeria and remains active in lobbying to ensure that the finances raised are not just secured for healthcare but are channelled specifically NCD control, instead. Having pushed the global surgery agenda to the launch of an NSOANP, global surgery champions in Nigeria may draw some lessons from the NASR and form a broad-based coalition to mobilize resources required to translate the plan into practice. Given the crucial role of surgery in treating non-communicable diseases, an alignment with NASR’s objectives may strengthen the opportunities of securing the sin tax revenues for healthcare, while pursuing evidence-based dialogue on health system priorities.

Our study has revealed the limited appreciation of the ripple effect of investments in surgical systems on the entire healthcare system, not just among non-health stakeholders but also within the clinical community. Because the surgical system is a microcosm of the wider healthcare system, sharing the same building blocks of infrastructure, workforce, service delivery, information management and financing, investing and strengthening surgical systems will likely benefit all other aspects of clinical care. There is a need for better advocacy to use available evidence to advance the cause of surgery among the non-surgery counterparts as a win–win for all.

Most participants agreed with the need to prioritize primary healthcare and reinvest money collected via sin taxes into strengthening primary care services. This consensus within the medical community and the broader health sector, along with the recent government transition in Nigeria, presents a policy window for advocates to challenge the lack of investment into surgical care. As Kingdon described it, policy change is likely to happen when the three streams—‘problem’ (lack of robust financing in primary healthcare), ‘policy’ (committing funds generated through sin taxes for healthcare), and politics (government transition) converge (Alcayaga *et al*., 2016). We therefore observe an opportunity for (additional) selected surgical procedures to be integrated into primary healthcare services, so that they can be effectively supported, in line with the 2015 World Health Assembly resolution 68.15 that recognized essential surgery and anaesthesia care as core components of universal health coverage (Federal Ministry of Health and Social Welfare of Nigeria, 2019).

There is a need to review the approaches to national surgical plans, which have been promoted to facilitate surgical system improvement. Past experiences from across LMICs have shown that plans are often financially intimidating, effectively rendering them infeasible, even though earlier estimates suggested that only modest per capita investments will be required in sub-Saharan Africa (Jumbam *et al*., 2019). The 17 billion dollars required for Nigeria’s NSOANP represents 35% of the total 2022 budget for the country. Our findings highlight the need for a change of approach in the structuring and financing of the plans, even while exploring other means to expand the fiscal space for surgery. To achieve this however, there is a need for evidence-based priority setting based on needs identified in the baseline assessment. Prioritization of the input requirements must align with a better understanding of the surgical production function (Zhang *et al*., 2022).

From the insights provided in the study, several recommendations could be derived regarding the improvement of financing of healthcare generally and surgical care specifically in Nigeria.

- Ideas: The FMOHSW should explore all possible avenues for expanding the fiscal space for health in Nigeria, including engaging national budgeting institutions for increment of health allocation to at least 15% in line with the Abuja Declaration; expanding the sin tax policy beyond SSBs; exploring other innovative financing options like aviation and tourism taxes; and pursuing full implementation of the 2022 NHIA. Global surgery scholars and champions within and outside Nigeria must collaborate to generate and leverage compelling evidence to promote the fortunes of surgery in the health fiscal space in Nigeria.
- Institutions: This study emphasizes the importance of inter-ministerial and inter-governmental engagement in advancing a particular agenda in the national polity. The FMOHSW in collaboration with the medical community and the CSOs must intensify official and unofficial engagement of the broader stakeholders particularly the Presidency, Ministry of Finance, the legislators, the state governors who more often than not determine the state representatives at the National Assembly and the leadership of the political parties, to amend the Finance Act to ring-fence the revenues from SSB tax for health. Moreover, internal heath system frameworks for accountability and probity must be strengthened, and general anti-corruption entities such as the Economic and Financial Crimes Commission (EFCC) and the Independent Corrupt Practices Commission (ICPC) must be empowered to effectively discharge their mandates to optimize judicious use of available resources within the health system. Demonstration of efficiency in the use of the presently available resources in the health sector could add a strong impetus in the negotiations for ear-marking the sin tax revenues for healthcare.
- Stakeholder interest and power: The FMOHSW must continue to identify and exploit points of common interest in advancing the health systems objective of improved financing for UHC, including using evidence to demonstrate to the Ministry of Finance and other stakeholders the broader impact of investments in health on other sectors such as education (Suhrcke and de Paz Nieves, 2011), national productivity (Tompa, 2002) and overall macro-economic development (Ashraf *et al*., 2008). Similarly, global surgery advocates should frame their arguments in the health space to ride on the shared interests with other stakeholders such as in primary healthcare. Meanwhile, it is important that any inter-professional rivalry within the medical community gives way for inter-professional synergy geared towards the common purpose of maximizing health system resources. Rather than subjective personal interests, resource allocation within the health system should be based on evidence. This requires sectoral capacity development in such areas as health technology assessment, enabling utilization of scientific tools like multi-criteria decision analysis (MCDA) (Diaby *et al*., 2013) and evidence-based deliberative processes to (EDPs) (Baltussen *et al*., 2022; Oortwijn *et al*., 2022) in priority setting and resource allocation decisions to maximize societal welfare gains from available resources.

### Strengths and limitations

This is the first study to empirically assess the potential for financing a national surgical plan using sin tax. The modelling relied on publicly available data, rather than information from the household consumption component of the 2018/2019 National Living Standards Survey, conducted by the National Bureau of Statistics (NBS) of Nigeria, which was not immediately accessible for use in this study. In addition, use of longitudinal price data from NBS would have permitted a more refined estimate of prices and price elasticities. However, we employed a set of sensitivity analyses to address uncertainties in our estimates, offering a robust and transparent overview on the impact of the SSB tax in Nigeria.

We conducted interviews amongst key stakeholders to learn about the potential for allocating funding to the NSOANP in Nigeria. The qualitative analysis would have benefitted from further engagement of law makers, Ministry of Finance representatives and industry representatives who were not captured in our study. However, we conducted a document analysis that provided useful insights into the perspectives and leanings of these stakeholders, further strengthening the key findings of our study.

The scope of our work was restricted to SSB tax, discounting the opportunities for revenue generation from other sin taxes such as those levied on tobacco and alcohol. It is likely that a combination of sin taxes on highly consumed products will not only provide substantial funds to support the NSOANP, but also other worthwhile health initiatives. Future research will be needed to model revenue gain across a multitude of target commodities and tax rates to offer policymakers information for evidence-based decision-making.

### Conclusion

At its current rate, the Nigerian SSB tax can only generate a fraction of the funding required to finance the NSOANP and is unlikely to incentivize significant changes in consumer behaviour to switch to healthier drinks options. Stakeholders should support the government efforts to adjust the tax structure to 20% ad valorem in line with WHO recommendations, while exploring other innovative options for expanding the fiscal space for health. There are currently no concrete frameworks earmarking any SSB tax revenue for healthcare, rendering funds susceptible to diversion to other non-health national needs, as the allocation of the funds is left to the priorities of national budget holders. Even where the funds are secured for the healthcare system, there appears to be insufficient momentum to tilt the consensus towards surgery specifically. NCD campaigners and surgery campaigners must work together to see that tax funds are secured exclusively for healthcare, and surgery advocates must intensify efforts towards better education of all stakeholders on the national benefits of investments in surgery.

## Supplementary Material

czae021_Supp

## Data Availability

Data are available from the corresponding author upon reasonable request.

## Reflexivity Statement

Among the research team, two authors are wholly based in Nigeria, the country of study, while six are based abroad. The lead author, though not based in Nigeria, is a Nigerian and had trained and practiced as medical doctor in Nigeria. With regards to gender, the team has an equal balance of male and female researchers (four versus four). Furthermore, the team comprised four senior researchers, one mid-level researcher and three junior researchers.
