# Supplementary material for: Can sugar taxes be used for financing surgical systems in Nigeria? A mixed-methods political economy analysis
Source: Health Policy Plan. 2024 Mar 29;39(5):509–18. doi: 10.1093/heapol/czae021 (PMC11095260; doi:10.1093/heapol/czae021)
Supplement: czae021_Supp [file czae021_supp.zip › suppl_data/Supplemental material 1_Data requirement and Sources.docx]

Supplementary material 1: Data requirements and sources

| S/N | Variable | Source | Remarks |
| --- | --- | --- | --- |
| 1 | Baseline annual SSB consumption (in litres) | Document analysis |  |
| 2 | Percentage equivalent of 10 Naira/litre | Document analysis |  |
| 3 | Own-price elasticities of SSBs | Peer-reviewed literature | This is the responsiveness of changes in consumption of SSBs to changes in their price |
| 5 | Pass-on rate | Peer-reviewed literature | This is the proportion of the tax that is passed on to consumers in the form of higher retail prices |
| 6 | Population | United Nations Population Prospects 2022 |  |
| 7 | Inflation projection | CBN and Statista |  |

Abbreviation: CBN, Central Bank of Nigeria; SSB, Sweetened and sugary beverages
