# Supplementary material for: Can sugar taxes be used for financing surgical systems in Nigeria? A mixed-methods political economy analysis
Source: Health Policy Plan. 2024 Mar 29;39(5):509–18. doi: 10.1093/heapol/czae021 (PMC11095260; doi:10.1093/heapol/czae021)
Supplement: czae021_Supp [file czae021_supp.zip › suppl_data/Supplemental material 2_Interview respondents_11Jan.docx]

**Supplementary material 2: Interview respondents**

| S/N | Respondent and background | Number |
| --- | --- | --- |
| 1 | Policymaker, federal (PM1) | 2 |
| 2 | Policymaker, state (PM2) |  |
| 3 | Civil society organisation/sugar tax campaigner (CSO1) | 2 |
| 4 | Civil society organisation/public health campaigner (CSO2) |  |
| 5 | Medical professional, surgeon, global surgery advocate (MP1) | 6 |
| 6 | Medical professional, internal medicine physician (MP2) |  |
| 7 | Medical professional, anaesthesiologist (MP3) |  |
| 8 | Medical professional, paediatrician (MP4) |  |
| 9 | Medical professional, obstetrician and gynaecologist (MP5) |  |
| 10 | Medical professional, public health physician (MP6) |  |
|  | Total | 10 |
