# Supplementary material for: Can sugar taxes be used for financing surgical systems in Nigeria? A mixed-methods political economy analysis
Source: Health Policy Plan. 2024 Mar 29;39(5):509–18. doi: 10.1093/heapol/czae021 (PMC11095260; doi:10.1093/heapol/czae021)
Supplement: czae021_Supp [file czae021_supp.zip › suppl_data/Supplemental materials 3_Question guide.docx]

**Supplemental materials 3: Question guide**

What is your view about the financing of healthcare in Nigeria?

What are the opportunities for mobilising resources for health?

What is your view on the recently introduced sin tax of 10N/l on SSBs?

Can this be implemented? Is it feasible?

What would be the barriers and obstacles to the implementation of this policy?

What is your view on the level it was placed ie 10N/l? To what extent can that deter consumption of SSBs? How much could be generated from this?

What is the possibility of channelling the resources raised to health strictly?

What could be the facilitating and militating factors against such moves?

Within the health system, what are the chances of channelling the resources to surgery specifically?

What are the facilitators and obstacles to such initiatives?

Can the players in the health sector achieve a consensus to dedicate the funds to NSOANP?

What are the priorities within the NSOANP?

For surgery to get into the mainstream of the fund, how do you think the messaging should be shaped/framed to achieve the desired results.

How do you think the money should be allocated in the health sector? What do you consider the priorities in the health sector?

What do you think about expanding this to alcohol and cigarettes?

What about phone call tariff?
